# CircDUSP22 Attenuates the Ferroptosis of Prostate Cancer Cells *via* miR-18a-5p/SLC7A11/GPX4 Signaling

**DOI:** 10.2174/0113862073324077240624094140

**Published:** 2024-07-11

**Authors:** Hua Jiang, He Zhang, Songsong Jiang

**Affiliations:** 1 Department of Urology, the Fifth Affiliated Hospital of Zunyi Medical University (Zhuhai Sixth People's Hospital), Zhuhai, China

**Keywords:** CircDUSP22, ferroptosis, prostate cancer, SLC7A11, *in vitro*

## Abstract

**Background:**

According to current worldwide cancer data, Prostate Cancer (PC) ranks as the second most common type of cancer and is the fifth leading cause of cancer-related mortality among men worldwide. PC in China has the 10th highest number of new cases and the 13th highest fatality rate, both of which show an ongoing annual increase. One of the significant challenges with prostate cancer is the difficulty in early detection, often resulting in diagnosis at intermediate or late stages, complicating treatment. Although hormonal therapy is initially successful in controlling the progression of prostate cancer, almost all tumors that respond to hormones eventually transform into Castration-resistant Prostate Cancer (CRPC) within 18-24 months of hormonal therapy. This poses clinical difficulties due to an absence of successful therapeutic approaches. Therefore, understanding the fundamental mechanisms of prostate cancer development, identifying effective therapeutic targets, and discovering reliable molecular biomarkers are crucial objectives.

**Methods:**

CircRNA expression in plasma was assessed in 4 samples obtained from patients with Benign Prostatic Hyperplasia (BPH), and PC was detected through microarray probes. Statistical analysis of the expression of circDUSP22 and clinicopathological features was conducted. The investigation of target genes was conducted using luciferase reporter assays and bioinformatics analysis. The expression levels of circDUSP22, miR-18a-5p, and Solute Carrier Family 7 member 11 (SLC7A11) were assessed using a quantitative Real-time Polymerase Chain Reaction (qRT-PCR) assay. Cell invasion, migration, colony formation, and proliferation were evaluated using Transwell, wound healing, colony formation, and CCK-8 assays, respectively. RNA Immunoprecipitation (RIP) and dual-luciferase reporter assays were used to examine the connections among circDUSP22, miR-18a-5p, and SLC7A11. The impact of circDUSP22 on the expression of ferroptosis-related proteins, specifically SLC7A11, as well as its effects on Fe2+ and ROS were also examined.

**Results:**

In both plasma samples and PCa cell lines, there was a substantial elevation of circDUSP22 and SLC7A11 expression and a decline in miR-18a-5p expression. Suppression of circDUSP22 significantly impeded the migration, invasion, and proliferation of PC cells *in vitro*. The target gene of miR-18a-5p, SLC7A11, was found to be upregulated as an effect of circDUSP22's competitive binding to miR-18a-5p. Cellular experiments demonstrated that interference with circDUSP22 expression in DU145 and PC-3 cells led to increased ferroptosis and decreased SLC7A11 expression. The modulation of prostate cancer cell proliferation was reversed by either overexpressing SLC7A11 or inhibiting miR-18a-5p in response to the silencing of circDUSP22.

**Conclusion:**

The circDUSP22 has been found to have a substantial effect on the development of ferroptosis in PC. It has been observed to influence the formation and evolution of this disorder by affecting the miR-18a-5p/SLC7A11 signaling pathway.

## 1 INTRODUCTION

PC is the primary malignancy that is diagnosed most frequently and ranks as the fourth leading cause of cancer-related deaths among men globally [1]. It has emerged as a significant health threat among older men. In recent years, significant progress has been made in the treatment of PC through the use of chemotherapy [2], immune checkpoint inhibitors [3], and bone-targeted drugs [4]. It is intriguing that among the numerous factors influencing the development of PC, recent studies have revealed that the gut and urogenital microbiomes may play a significant role in the pathogenesis of PC [5]. However, early diagnosis and the pathogenesis of PC remain unclear. Therefore, it is of utmost importance to thoroughly understand the molecular mechanisms underlying the development of PC to identify novel biomarkers and establish a theoretical foundation for the treatment of this disease.

Ferroptosis is a recently identified type of programmed cell death that differs from traditional processes, like apoptosis or necrosis, in terms of appearance, biochemistry, and physiology. Ferroptosis is reliant on lipid peroxidation [6]. The onset of ferroptosis involves complex biological and biochemical processes triggered by disturbances in iron, lipids, and antioxidants. However, the specific roles and effects of these factors on cancer development and metastasis remain unclear. Studies have demonstrated the mutual influence between ferroptosis and numerous traditional cell signaling pathways within cancers. One example is the activation of the AMP-activated Protein Kinase (AMPK)-Acetyl-CoA Carboxylase (ACC)-Polyunsaturated Fatty Acid (PUFA) pathway. This system, which plays a role in energy metabolism, decreases the production of PUFAs, therefore preventing ferroptosis [7]. The lactate produced by tumor metabolism not only offers a favorable environment for tumor spread, but it can additionally inhibit ferroptosis in tumor cells by activating the Monocarboxylate Transporter 1 (MCT1) and Hydroxy-carboxylic Acid Receptor 1 (HCAR1). The Stearoyl-CoA Desaturase 1 (SCD1) and Sterol Regulatory Element Binding Protein 1 (SREBP1) pathways are responsible for promoting tumor growth and metastasis [8]. Recent research suggests that adjacent cells can hinder ferroptosis in tumor cells through E-cadherin-mediated interactions that activate the NF2 and Hippo signaling pathways [9]. Moreover, SLC7A11 promotes the absorption of cystine, which boosts the production of Glutathione (GSH) to minimize the accumulation of lipid peroxidation products and thus inhibit ferroptosis [10, 11].

Cysteine is a crucial amino acid in organisms. Apart from its role as a free radical scavenger and antioxidant, it also acts as a metabolic enhancer. Additionally, it plays a significant role in protein synthesis, posttranslational modifications, and other cellular processes [12]. The demand for cysteine in normal cells can be met through either the transsulfuration pathway or the degradation and recycling of proteins [13]. On the other hand, cancer cells mainly acquire cysteine from outside their environment *via* SLC7A11. This protein acts as the smaller component and is highly attractive for transporting cystine and glutamate. Together with its larger subunit (SLC3A2), it forms the Xc- system. Intracellularly, cystine is promptly converted to cysteine [14]. The transport of cystine facilitated by SLC7A11 is essential for inhibiting oxidative processes and maintaining cell viability during periods of oxidative stress [15]. Growing evidence indicates that SLC7A11 is significantly upregulated in various malignancies and is closely associated with tumor metabolism. The expression of SLC7A11 can be triggered in stressful situations, including oxidative stress, metabolic stress, and amino acid deprivation. The key transcription factors responsible for regulating the expression of SLC7A11 are NRF2 and ATF4 [16, 17]. Studies have shown that triptolide binds directly to SLC7A11, resulting in deactivation of the SLC7A11/glutathione peroxidase 4 signaling pathway, which is involved in ferroptosis and associated with triptolide-induced cardiac toxicity [18]. In studies on acquired sorafenib resistance in liver cancer, ABCC5 has been identified as a crucial regulator in human hepatocellular carcinoma cells. It stabilizes the SLC7A11 protein, increases intracellular GSH levels, and decreases lipid peroxide accumulation, thereby inhibiting ferroptosis [19].

Furthermore, the expression of SLC7A11 is controlled by a range of epigenetic factors, including miRNAs, circRNAs, and lncRNAs [20, 21]. Immunotherapy and radiotherapy, which are commonly used to treat cancer, can partially trigger ferroptosis by modifying the expression of SLC7A11 [22]. Nevertheless, more research is necessary to thoroughly understand the intricate regulation of SLC7A11 expression and transporter activity, as well as its unique function in controlling ferroptosis in prostate cancer cells.

Due to its covalently closed loop structure, circRNA is more stable compared to other types of RNA [23, 24]. An increasing body of research [25-28] indicates a close association between circRNAs and prostate cancer. Studies have shown that circ_0076305 can affect the expression of PGK1 by sequestering miR-411-5p. Inhibiting the expression of circ_0076305 in animal models suppressed the growth of prostate tumors *in vivo* [29]. Circ_0063329 is significantly downregulated in prostate cancer cells and tissues, and it can inhibit the proliferation and metastasis of prostate cancer by modulating the miR-605-5p/TGIF2 axis. Additionally, circular RNAs also play a crucial role in radioresistance in prostate cancer [30]. Silencing circ-ABCC4 has been found to impair the survival, proliferation, invasion, and radioresistance of PCa cells, leading to apoptosis. Inhibiting the expression of circ-ABCC4 exacerbates the inhibitory effect of radiation-induced growth suppression on xenografts [31]. Furthermore, research has uncovered a novel circular RNA, circ_0004087, which modulates the spindle assembly checkpoint through the SND1/MYB/BUB1 axis, affecting mitotic error correction mechanisms and thereby regulating chemosensitivity to docetaxel [32]. However, the current understanding of the role of circRNAs in regulating the sensitivity of prostate cancer cells to ferroptosis remains limited.

This study has mainly explored the regulatory function of circDUSP22 in modulating the expression of SLC7A11, thereby impacting ferroptosis in prostate cancer cells. It can contribute to the increasing research on the role of circular RNAs in regulating ferroptotic mechanisms in prostate cancer.

## 2 METHODOLOGY

### 2.1 Patients and Samples

At the Fifth Affiliated Hospital of Zunyi Medical University in Zhuhai, China, 36 patients with prostate cancer provided plasma and tissue samples, which were then preserved at -80°C. The inclusion criterion was patients diagnosed with prostate adenocarcinoma through transrectal ultrasound-guided prostate biopsy, and having no history of radiotherapy, chemotherapy, or endocrine therapy. The exclusion criteria included patients with concurrent tumors in other locations or secondary tumors of the prostate. Plasma samples collected from 24 patients diagnosed with Benign Prostatic Hyperplasia (BPH) were utilized as the control cohort.

### 2.2 CircRNA Array Analysis

The human circRNA Array v2 system from Arraystar was used to evaluate the specimens. Following the quantification of total RNA with a microarray hybridization technique and NanoDrop ND-1000 instrument, sample preparation was conducted in accordance with Arraystar's standard operating procedures. Briefly, RNase R digestion was performed on total RNA to eliminate linear RNAs and increase the abundance of circular RNAs. Following that, fluorescent cRNAs were transcribed from the enriched circular RNAs *via* amplification and random priming using the Super RNA Labeling Kit (Arraystar Inc., USA). Using the Agilent G2505C scanner, the labeled cRNAs were hybridized onto the human circRNA array (v2; 8x15K; Arraystar Inc., USA). The data analysis was performed utilizing Agilent Feature Extraction software (version 11.0.1.1) and the limma module of the R software was utilized to normalize quantiles and perform additional data processing.

### 2.3 Cell Culture

The PC cell lines, DU145 and PC3, and the nonmalignant epithelial prostate cell line RWPE-1 were obtained from the Chinese Academy of Sciences' Cell Bank of Type Culture Collection in Shanghai, China. These cell lines were maintained at 37°C in a humidified environment with 5% CO_2_ in RPMI-1640 medium containing 10% heat-inactivated FBS.

### 2.4 qRT‒PCR Assay

The extraction of total RNA was performed using TRIzol reagent according to the directions provided by the manufacturer (Life Technologies, CA, USA). The RNA samples were examined for concentration and purity using a NanoDrop 2000 spectrophotometer (Thermo Scientific, MA, USA). This was done by measuring the A260/A280 ratio and confirming that the readings were within the recommended range of 1.8–2.1 Subsequently, PrimeScript Reverse Transcription (RT) reagent (RR047A, Tokyo, Japan) was used to convert 5 µg of RNA into complementary DNA (cDNA). The qRT-PCR analysis was conducted employing an ABI Prism 7500 Fast Real-time PCR machine (Applied Biosystems, CA, USA) and the SYBR Premix Ex Taq Kit (Takara, Japan). Through specific primers, the expression of circDUSP22 was quantified, with the Glyceraldehyde 3-Phosphate Dehydrogenase (GAPDH) gene serving as the internal reference. Using the 2-∆∆Ct technique, the relative expression levels of circDUSP22 were ascertained.

### 2.5 Actinomycin and RNase R treatment

RNA polymerase-mediated RNA chain elongation was consistently inhibited with 2 mg/ml actinomycin D (Sigma, MO, USA) in order to validate the circular structure of circDUSP22. A total of 10 U of RNase R (Epicenter Technologies, WI, USA) was applied to 2 μg of total RNA from DU145 cells at 37°C for 30 minutes. Following processing, qRT-PCR was utilized to examine the RNA samples.

### 2.6 RNA Fluorescence *In situ* Hybridization (FISH)

For cell-based Immunofluorescence (IF) analysis, DU145 cells were fixed in 4% paraformaldehyde. The DU145 cells were prehybridized in 1× PBS/0.5% Triton X-100 and then hybridized with the circDUSP22 FISH probe in a hybridization buffer overnight at 4°C. Following complete washing, 4',6-Diamidino-2-phenylindole (DAPI) was used as a counterstain for the nuclei. We used a fluorescent microscope (Carl Zeiss, LSM 510, Germany) for imaging and analysis.

### 2.7 Colony Formation Assay

An assay for colony formation was utilized to measure cell proliferation. The treated cells were planted at 10,000 cells/well density in 6-well plates, and they were kept in a complete medium for 10 days at 37°C and 5% CO_2_. On a daily basis, the colonies were examined, quantified, and preserved in paraformaldehyde (4%) and then examined under a light microscope (Olympus, Tokyo, Japan) using 0.5% crystal violet staining. Three duplicates of this experiment were carried out.

### 2.8 Cell Counting Kit-8 (CCK-8) Assay

In order to evaluate the growth and survival of prostate cancer cells, the cells that were subjected to treatment were placed in 96-well plates with a 1×104 density of cells/well. These cells were then cultured for different periods. Afterward, the CCK-8 reagent from Sigma‒Aldrich was introduced to each well. The plates were subsequently placed in an incubator at a temperature of 37°C with a CO_2_ concentration of 5% for 2 h. A microplate reader (EL×808; Bio Tek, USA) was used to measure the absorbance at 450 nm. Each experiment was repeated three times.

### 2.9 5-Ethynyl-2-deoxyuridine (EdU) Incorporation Assay

The Cell-LightTM EdU DNA Cell Proliferation Detection Kit (RiboBio, Shanghai, China) was used to perform the EdU incorporation experiment. After a 2h incubation at 37°C with 5% CO_2_, transfected cells were independently placed in the wells of 96-well plates containing EdU medium diluent. The cells were fixed for 20 minutes with 4% paraformaldehyde, stained for 30 minutes using Apollo Dye Solution (RiboBio, China), and counterstained for an extra 30 min at room temperature using DAPI. This experimental procedure was repeated in triplicate.

### 2.10 Transwell Assay

The assessment of cellular invasion and migration was conducted using the Transwell assay. After 48 h of transfection, DU145 and PC-3 cells were placed in a medium without serum and then placed in the upper chamber. The RPMI 1640 medium containing 10% FBS was added to the lower chambers, which acted as a chemoattractant. After 24 hours of incubation, the cells that had migrated and invaded were treated with methanol to fix them, stained with a solution containing crystal violet (0.1%), and then observed through a light microscope manufactured by Olympus (Tokyo, Japan).

### 2.11 Western Blot Assay

Cell lysates were produced using Radioimmunoprecipitation (RIPA) lysis buffer for Western blot analysis. The total proteins were transferred onto PVDF (Polyvinylidene Fluoride) membranes following their separation using 10% SDS-polyacrylamide gel electrophoresis. Subsequently, these membranes were probed using particular primary antibodies, specifically β-actin (Abcam, UK, ab8227) and SLC7A11 (Abcam, UK, ab37185). Following this, a secondary antibody labeled with horseradish peroxidase (1:5000, Abcam, UK) was incubated for 1 h at 37°C. The blots have been observed using an enhanced Chemiluminescence (ECL) detection kit (Beyotime, China).

### 2.12 Dual-luciferase Reporter Assay

For this experiment, the Dual-Luciferase® Reporter (DLRTM) experiment system was utilized. The pGL3 basic vector (Invitrogen, USA) was modified to contain the Wild-type (WT) and Mutant (MUT) miR-18a-5p binding site segments of the circDUSP22 or SLC7A11 3'UTR. PC3 cells underwent transfection with the miR-18-5p mimic, miRNA NC, and the vector. The luciferase activity of the transfected cells was measured 48 hours post-transfection.

### 2.13 RNA Pull-down Assay

In order to develop probe-coated beads for the circDUSP22 pull-down miRNA experiment, biotin-labeled circDUSP22 and oligonucleotide probes were combined with streptavidin magnetic beads in RIP buffer. These beads were then incubated with cell lysates, followed by washing and quantification of circDUSP22 and miRNAs using qRT‒PCR.

### 2.14 Detection of Intracellular Iron and ROS Activity

We plated 1×106 cells from each of the four designated culture groups into individual wells of a 12-well plate. Subsequently, the cells were cultured at 37°C in a 5% CO2 atmosphere for 12 h. Following the incubation period, cells from all groups were collected, and the concentrations of intracellular iron ions and ROS activity were measured according to the protocols. The concentrations of iron and Fe2+ were quantified using an Iron Assay Kit (Sigma, USA, MAK025), and the levels of ROS were examined using DCFH-DA staining (Sigma, USA, 4091-99-0, 287810).

### 2.15 Statistical Analysis

GraphPad Prism 6.0 was used to analyze the data, which have been displayed as mean ± SD. Inter-group comparisons were conducted using one-way Analysis of Variance (ANOVA), followed by LSD tests for pairwise comparisons when the variances were homogeneous and Tamhane's T2 test when the variances were unequal. SLC7A11 and miR-18-5p levels were associated using a Pearson's correlation analysis. A *P*-value of <0.05 was considered statistically significant.

## 3 RESULTS

### 3.1 CircDUSP22 found to be significantly expressed in PCa cell lines and patient plasma

**Fig. (1):**
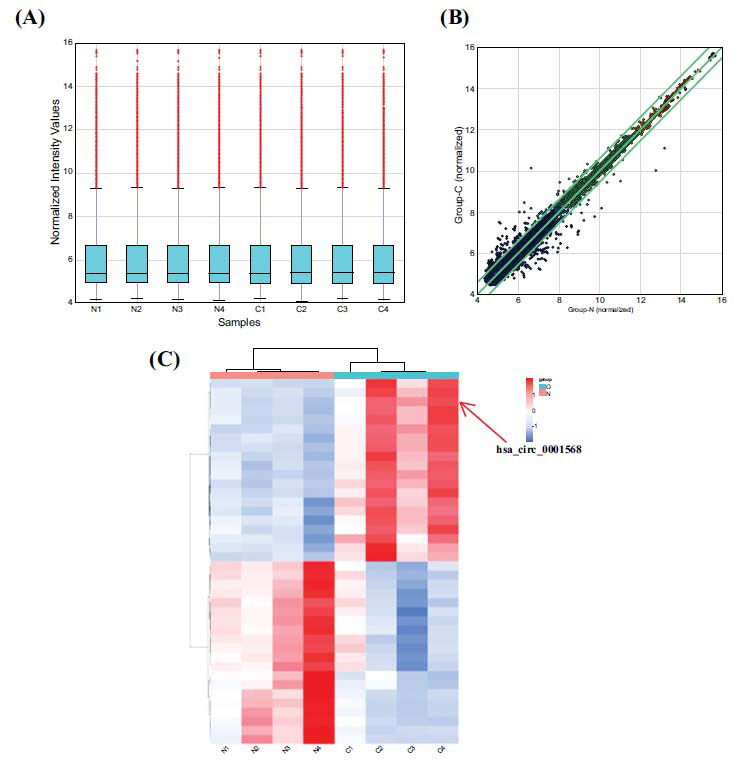
Identification of circRNAs with changed expression levels in the serum of individuals diagnosed with prostate cancer. (**A**). Standardization of serum samples. (**B**) Scatter plot illustrating the expression levels of circRNAs that are differently expressed. (**C**). Heatmap of the circRNA chip.

**Fig. (2):**
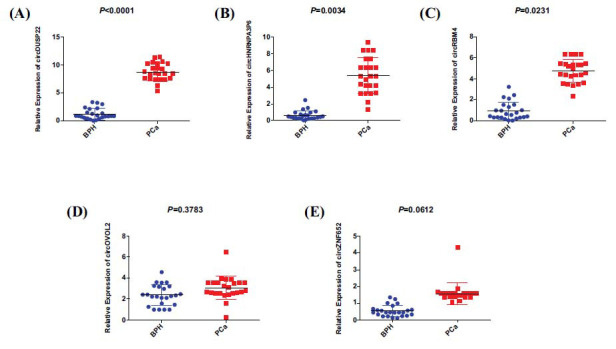
Five differently expressed circRNAs found using qRT-PCR in matched serum samples from 24 patients with benign prostatic hyperplasia and prostate cancer (n=24).

The Arraystar Human circRNA, Array (v.2) technology, was employed to examine plasma samples from 4 individuals with PCa and 4 patients with BPH in order to explore circRNAs that may be associated with the development of PC (Fig. **1a**-**c**). A heatmap showing the top 20 circRNAs that were upregulated and downregulated was created utilizing the R program (Fig. **1d**). Following that, qRT-PCR assay was conducted to assess the relative expression levels of these 5 circRNAs in a separate group of serum samples from 24 pairs of individuals with PCa and BPH. The findings indicated a statistically noteworthy variance in the expression levels of circHNRNPA3P6, circRBM4, and circDUSP22 between the sera of PCa patients and BPH patients (Fig. **2a**-**c**). In contrast, circZNF652 and circOVOL2 exhibited no notably elevated plasma expression levels in PCa patients compared to that in the plasma of patients with BPH. Notably, among these genes, circDUSP22 demonstrated the most substantial difference in its relative expression level (Fig. **2d**-**e**).

### 3.2 Characteristics of circDUSP22 in Prostate Cancer

**Fig. (3):**
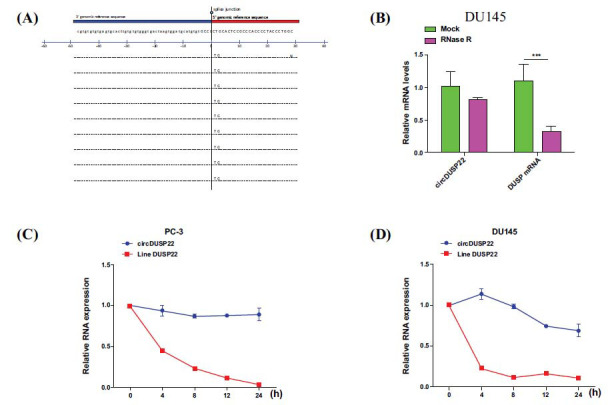
General biological characteristics of circDUSP22 in prostate cancer cells. (**A**). The circBase online website provides the splicing sites of circDUSP22. (**B**). The expression of circDUSP22 and DUSP22 mRNA in DU145 cells was evaluated by qRT-PCR after treatment with RNase R. **(C**). Actinomycin D-treated PC3 cells' circDUSP22 and DUSP22 mRNA expressions were analyzed using qRT-PCR. (**D**). DUSP22 and circDUSP22 mRNA expression in DU145 cells following actinomycin D treatment was analyzed using qRT-PCR; 18S RNA served as an internal control.

A series of experimental validations and localization assays, which included RNase R digestion experiments, actinomycin D inhibition assays, and nuclear-cytoplasmic RNA fractionation experiments, were conducted to confirm the closed-loop structure of circDUSP22. The findings demonstrated circDUSP22 to be mainly present in the cytoplasm of prostate cancer cells, suggesting a high probability of the involvement of ceRNA mechanism (Fig. **3a**). The RNA digestion assay utilizing the RNase R enzyme illustrated the relative resistance of circDUSP22 to degradation by RNAases in comparison to its linear precursor gene DUSP22 (Fig. **3b**). Furthermore, actinomycin D therapy had little effect on circDUSP22 expression (Fig. **3c** and **d**). These collective experimental results preliminarily confirmed the circular conformation of circDUSP22 and its stability within plasma samples. These results provide a solid foundation for our future research and indicate that circDUSP22 may become a valuable biomarker for prostate cancer patients’ diagnosis and prognosis.

### 3.3 *In Vitro* PCa Cell Proliferation, Migration, and Invasion were all Decreased by circDUSP22 Knockdown

**Fig. (4):**
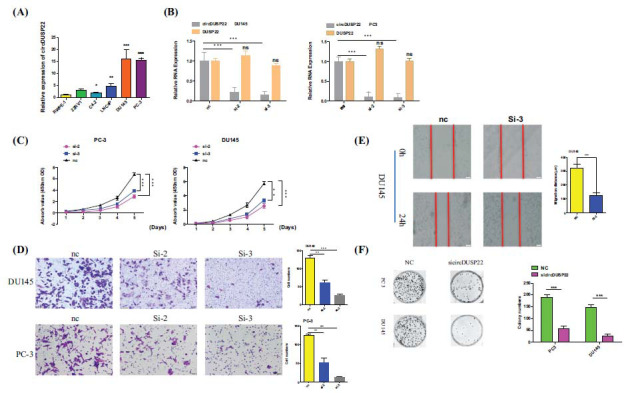
No increase in prostate cancer cells *in vitro* when circDUSP22 is silenced. (**A**). Using qRT-PCR, the relative expression levels of circDUSP22 were determined in 5 PC cell lines (PC-3, LNCaP, DU145, 22RV1, and C4-2), as well as in the normal human epithelial cell line RWPE-1. (**B**). DUSP22 and circDUSP22 expressions in DU145 and PC-3 cells transfected with two distinct circDUSP22 siRNAs were investigated using qRT-PCR to confirm the effectiveness of the interference. (**C**). Growth curves of PC-3 and DU145 cells following transfection with circDUSP22 siRNA, as determined by the CCK-8 assay. (**D**). Following circDUSP22 knockdown, the migratory potential of DU145 and PC-3 cells was assessed using transwell assays. (**E**). A wound-healing assay was used to determine the migratory capacity of PC cells. (**F**). After circDUSP22 was silenced, the plate colony formation test was used to evaluate the PC cells' capacity for proliferation (* P <0.05, ** P <0.01, *** P <0.001).

For a comprehensive analysis of the biological function of circDUSP22 in PCa cells, the study initially carried out a quantitative analysis to measure its natural expression levels in both PCa cells and normal prostate epithelial cells using qRT-PCR. After identifying the DU145 and PC-3 cell lines exhibiting relatively elevated circDUSP22 expression, 2 siRNAs were custom-designed to target the backsplice junction. Subsequent functional assays revealed a notable increase in circDUSP22 expression in PCa cell lines compared to noncancerous prostate epithelial cells (Fig. **4a**). The silencing of circDUSP22 in DU145 and PC-3 cells using siRNA effectively suppressed its expression, as verified by qRT‒PCR (Fig. **4b**). As demonstrated by CCK-8 experiments that showed considerably reduced proliferation rates in silenced DU145 and PC-3 cells, this knockdown significantly reduced the *in vitro* proliferative potential of PCa cells (Fig. **4c**), suggesting that increased circDUSP22 expression may contribute to PCa progression through enhanced cellular proliferation.

The effect of circDUSP22 on the migratory properties of PCa cells was assessed with the help of scratch wound healing tests and transwell migration assays. The findings suggested circDUSP22 to be involved in PCa metastasis since it significantly reduced the migratory potential of PCa cells when compared to controls (Fig. **4d** and **e**). In addition, a clonogenic experiment was performed to assess PCa cells' capacity to form colonies following circDUSP22 interference. After the circDUSP22 knockdown, DU145 and PC-3 cell colony numbers substantially dropped in comparison to the control group (Fig. **4f**).

### 3.4 DDX39A Mediates the Nucleocytoplasmic Export of circDUSP22

**Fig. (5):**
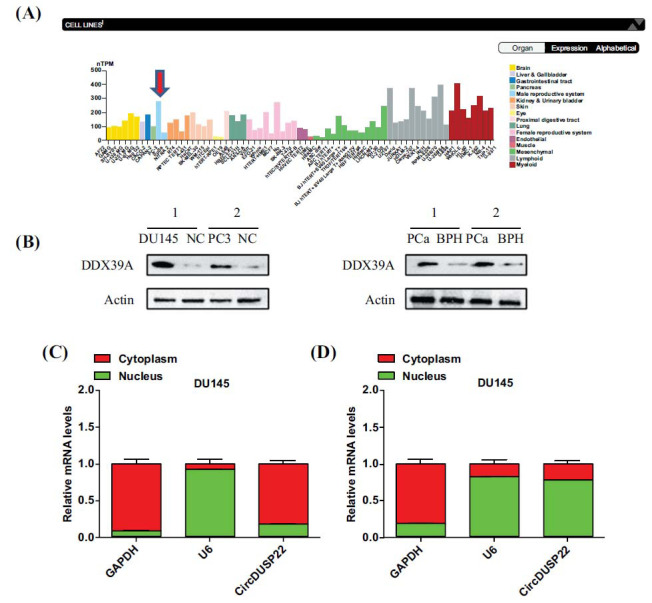
Online prediction and differential expression of DDX39A. (**A**). The Human Protein Atlas, an online bioinformatics platform, indicated a significant expression of DDX39A in the PCa cell line PC3. (**B**). DDX39A expression was substantially greater in PCa cell lines compared to normal control cells. Additionally, the expression in prostate cancer tissues was higher in comparison to patients with benign prostatic hyperplasia. (**C**). CircDUSP22 was primarily expressed in the cytoplasm of DU145, a cell line associated with prostate cancer. (**D**). After silencing DDX39A expression, qRT‒PCR analysis revealed the nuclear retention of circDUSP22.

Through online predictions on the Human Protein Atlas website, we observed high expression of DDX39A in the PC cell line PC3 (Fig. **5a**). The PC cell lines, DU145 and PC3, were next subjected to Western blot analysis, which verified the DDX39A upregulation in both cell types relative to the corresponding normal control cells. Similarly, prostate cancer tissues have been found to have higher amounts of DDX39A than tissues of benign prostatic hyperplasia, according to Western blot assays (Fig. **5b**).

Moreover, this study has revealed circDUSP22 to be mainly expressed in the cytoplasm of DU145 cells generated from PC cells using nuclear-cytoplasmic separation and qRT-PCR. Due to this atypical expression pattern, we silenced DDX39A and repeated the nuclear-cytoplasmic fractionation and qRT‒PCR analyses. Subsequent investigations have revealed that upon DDX39A knockdown, circDUSP22 exhibited nuclear retention, as indicated by increased expression levels within the nucleus compared to those in the cytoplasm (Fig. **5c** and **d**).

### 3.5 CircDUSP22 Functions like a Sponge for miR-18a-5p, Controlling the Expression of SLC7A11

**Fig. (6):**
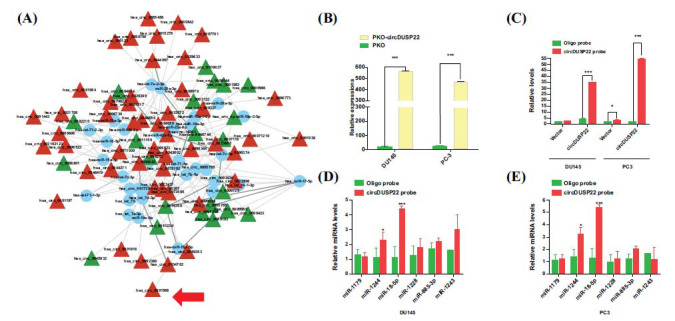
CircDUSP22 functions as a molecular sponge for miR-18a-5p. (**A**). Prediction and network construction of target genes of differentially expressed circRNAs predicted that circDUSP22 can act as a sponge to absorb miR-18a-5p. (**B**) Plasmids overexpressing circDUSP22. (**C**). The expression of the target circDUSP22 in the circDUSP22 probe cohort was substantially reduced compared to that of the control group. (**D, E**). miR-18a-5p was much more abundant in circDUSP22 in the two cell lines, according to qPCR analysis.

Regarding the regulatory mechanism, the study has suggested circDUSP22 to function as a molecular sponge for miR-18a-5p, modulating its activity. Through utilizing network analysis and computational prediction of downstream targets of dysregulated circRNAs, hsa-miR-18a-5p was identified as a probable candidate of circDUSP22 (Fig. **6a**). Experimental validation *via* luciferase assays supported this hypothesis, indicating an interaction between the two proteins. The RNA pull-down assays were performed in cells that had an increased expression of circDUSP22. These assays have confirmed the circDUSP22 probes to be bound explicitly to miR-18a-5p in the PC cell lines, DU145 and PC-3 (Fig. **6b** and **c**). Additional confirmation was obtained from dual-luciferase reporter experiments, in which the vector harboring the whole circDUSP22 sequence's luciferase activity decreased upon the introduction of the miR-18a-5p mimic. At the same time, no impact was observed on the mutated version lacking functional miR-18a-5p binding sites (Fig. **6d** and **e**). These findings have validated circDUSP22's capacity to sequester miR-18a-5p.

**Fig. (7):**
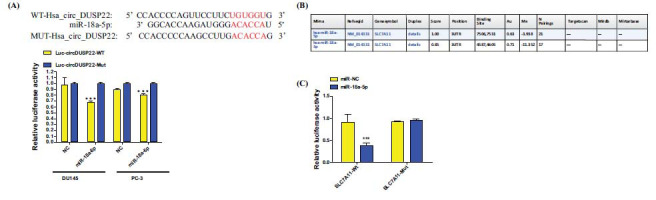
CircDUSP22 acts as a molecular sponge that sequesters miR-18a-5p and modulates the activity of SLC7A11. (**A**). The circDUSP22 WT and Mut luciferase reporter vectors were constructed for this study. Dual-luciferase reporter gene tests were conducted to assess luciferase activity. The results showed a decrease in luciferase activity when cells were co-transfected with circDUSP22-WT and miR-18a-5p mimics. (**B, C**). The target genes of miR-18a-5p were predicted using the bioinformatics websites, miRWalk and starBase. The transfection of miR-18a-5p significantly reduced luciferase activity in the WT SLC7A11 3'-UTR vector, but not in the mutant SLC7A11 3'-UTR vector, according to qRT-PCR data.

**Fig. (8):**
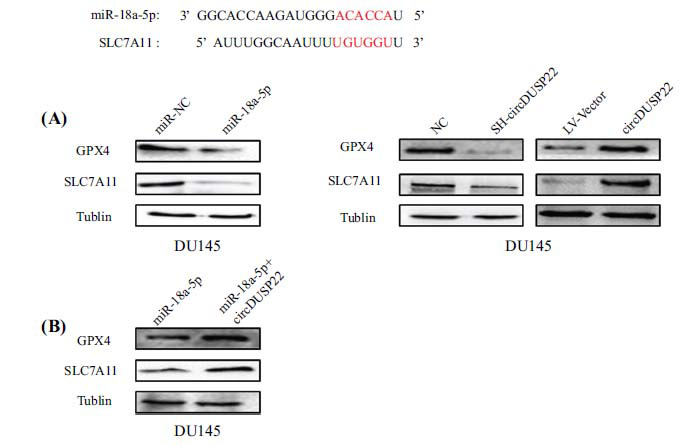
CircDUSP22 upregulates the expression of SLC7A11 by absorbing miR-18-5p. (**A**). miR-18a-5p overexpression suppressed the protein expression of SLC7A11, while circDUSP22 overexpression led to an increase in the protein expression of SLC7A11. (**B**). The SLC7A11 expression levels were substantially higher in cells transfected with miR-18a-5p+circDUSP22 compared to those transfected with miR-18a-5p alone, resulting in a concurrent elevation in GPX4 expression.

Subsequently, utilizing bioinformatics tools, such as miRWalk and starBase, the research team predicted the miR-18a-5p target genes. It was found to be bound to SLC7A11 due to robust support from AGO CLIP-Seq data and high predictive scores (Fig. **7a**). The interaction between the 3'-UTR of SLC7A11 and miR-18a-5p has been verified. The binding and regulatory effect of the miR-18a-5p on SLC7A11 mRNA has been validated through additional dual-luciferase reporter tests (Fig. **7b** and **c**). The Western blot studies revealed that the overexpression of miR-18a-5p led to a substantial decrease in the protein levels of SLC7A11 and GPX4 than the miR-NC group. Furthermore, investigations involving the manipulation of cell lines demonstrated that the increase in circDUSP22 expression caused an elevation in the levels of SLC7A11 and GPX4 proteins. In contrast, the reduction of circDUSP22 resulted in a decrease in their levels (Fig. **8a**).

In order to confirm the hypothesis that circDUSP22 acts as a sponge for miR-18a-5p in controlling the expression of SLC7A11, a set of transfection experiments were performed on DU145 prostate cancer cells. In addition, these cells were co-transfected or individually transfected with miR-18a-5p along with both miR-18a-5p and circDUSP22. Following transfection, SLC7A11 expression levels were quantified by Western blot analysis. The findings demonstrated that the coexpression of miR-18a-5p and circDUSP22 increased SLC7A11 expression significantly in comparison to the group transfected with miR-18a-5p alone. Concurrent alterations in GPX4 levels were also observed (Fig. **8b**).

**Fig. (9):**
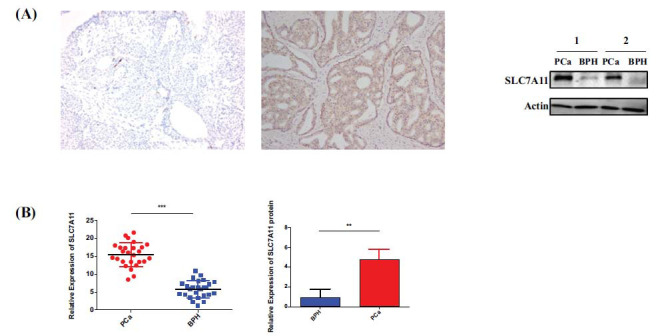
Expression of SLC7A11 in prostate cancer tissues. (**A**). Immunohistochemistry and Western blot analysis showed negative expression of SLC7A11 in benign prostatic hyperplasia tissues. Simultaneously, it was highly positively expressed in PC tissues, mainly in the cancer cells' cytoplasm. (**B**). The qRT‒PCR assay results.

Furthermore, to investigate the clinical relevance of these findings, immunohistochemical and Western blot analyses were conducted on BPH and PCa tissues. Histological evaluations revealed SLC7A11 immunostaining to either be absent or weakly positive in BPH tissues (Fig. **9a**). In contrast, it exhibited strong positivity in PCa tissues, predominantly localized within the cytoplasmic compartment of cancer cells. The statistical comparisons of SLC7A11 expression between BPH and PCa tissues revealed a statistically significant rise in the latter's levels (Fig. **9b**). This finding adds credence to the theory that circDUSP22, miR-18a-5p, and SLC7A11 may interact in a significant way during the initiation and development of prostate cancer.

### 3.6 CircDUSP22 Inhibits Ferroptosis in PCa Cells

**Fig. (10):**
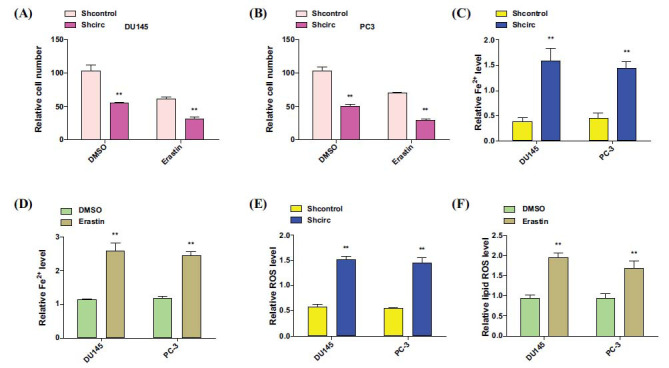
CircDUSP22 inhibits ferroptosis in prostate cancer cells. (**A** and **B**). The CCK-8 assay was utilized to quantify the activity of the PCa cell lines, DU145 and PC3. Following interference with circDUSP22 expression, the activity of PC3 and DU145 cells was substantially diminished during erastin administration. (**C-F**). Changes in the quantities of Fe2+ and ROS expression in PCa cells following various treatments.

To further elucidate the relationship between circDUSP22 and ferroptosis, we employed an experimental approach involving the manipulation of circDUSP22 expression. Subsequently, we examined the expression patterns of iron-dependent ferroptosis biomarkers, specifically ferrous iron (Fe2+) and Reactive Oxygen Species (ROS). Following the successful knockdown of circDUSP22, DU145 and PC3 cells were treated with erastin, a known ferroptosis inducer. Cell viability significantly decreased in both cell lines when circDUSP22 was depleted, and erastin therapy was applied, as shown by cytotoxicity studies (Fig. **10a** and **b**).

Moreover, upon silencing circDUSP22, we observed a notable increase in the intracellular levels of Fe2+ and ROS, both of which are indicative of ferroptosis processes (Fig. **10c**-**f**). These data suggest a potentially crucial role of circDUSP22 in mediating ferroptosis resistance in PC3 and DU145 prostate cancer cells, as its downregulation appears to enhance the induction of ferroptosis, as evidenced by the altered levels of key ferroptosis-related molecular markers.

## 4 DISCUSSION

According to recent research, ferroptosis is essential for a number of physiological and pathological processes related to malignancies of the urogenital system, such as tumor migration and mesenchymal transformation [33]. Specifically, it is crucial to the management of castration-resistant prostate cancer and the carcinogenic pathways of prostate cancer [34]. In pancreatic cancer cells, circ_WASF2 is significantly overexpressed, promoting pancreatic cancer cell proliferation by targeting the miR-634/GPX4 axis. This suggests that targeting circ_WASF2 expression could be a promising therapeutic strategy for pancreatic cancer treatment [35]. Studies on breast cancer have revealed that the overexpression of FOXQ1 in breast cancer cells leads to increased levels of circ_0000643 by binding to its host gene promoter region. Circ_0000643 acts as a sponge for miR-153, enhancing SLC7A11 expression and inhibiting ferroptosis in breast cancer cells [36]. On the other hand, circ_0087851 shows markedly reduced expression in colorectal tissues and cells. It acts as a tumor suppressor and inducer of ferroptosis in colorectal cancer by modulating the miR-593-3p/BAP1 axis, thus regulating ferroptosis in colorectal cancer [37]. Additionally, circular RNAs play a crucial role in the development of cisplatin resistance in various malignant tumors [38-41]. Specifically, circ_0000140 is significantly upregulated in oral squamous cell carcinoma cells. Knocking down circ_0000140 enhances the sensitivity of oral squamous cell carcinoma cell lines to cisplatin by modulating the miR-527/SLC7A11 signaling pathway, leading to increased ferroptosis [42]. Ferroptosis is a form of programmed cell death that is triggered by damage caused by lipid peroxidation and is dependent on the presence of iron. This process exhibits genetic, biochemical, and morphological distinctions from common cellular death mechanisms, including apoptosis, autophagy, pyroptosis, and necrosis. Moreover, it represents a novel mode of cell death characterized by distinctive biochemical features, including the involvement of iron, accumulation of lipid peroxides and reactive oxygen species, suppression of the cystine/glutamate antiporter, diminished synthesis of glutathione, and oxidation of reduced Nicotinamide Adenine Dinucleotide Phosphate (NADPH) [43]. The mechanism underlying ferroptosis involves free intracellular iron ions reacting with peroxides through the Fenton reaction, leading to further peroxidation of polyunsaturated fatty acids in biomembranes [44]. This process is regulated by several genes, including Heme Oxygenase 1 (HMOX1), which is related to iron metabolism; cytoplasmic adaptor proteins, which are involved in antioxidant metabolism; Glutaminase 2 (GLS2), which is associated with energy metabolism; and GPX4, which is pertinent to lipid metabolism.

The importance of ferroptosis in tumor growth and metastasis is underscored by research findings indicating that resistance to ferroptosis is a crucial trait of metastatic cancer cells. By inducing ferroptosis, the potent tumorigenic and metastatic activities of drug-resistant cancer cells can be diminished. These discoveries highlight the universal relevance of ferroptosis in tumor progression and metastasis. Metastatic cancer cells are shielded from ferroptosis in the circulation and premetastatic microenvironment by an antioxidant defense mechanism [45, 46]. These findings suggest that influencing cancer progression by selectively inducing or inhibiting ferroptosis can have a significant impact. Research focusing on circular RNAs that regulate cancer progression through targeted induction or inhibition of ferroptosis has garnered considerable attention. Among these, the most prevalent mechanism involves circular RNAs acting as competing endogenous RNAs (ceRNAs) to modulate ferroptosis levels in cancer. For example, circ0082374 and SLC7A11 reciprocally control their expression levels in non-small cell lung cancer cells. This regulation affects ferroptosis by means of circ0082374's sponge effect on miR-491-5p, which can either increase or decrease the capacity for tumor growth [47]. One solute carrier family member, SLC7A11, is essential for encoding the cystine/glutamate antiporter Xc system [48]. A critical function of the light chain subunit SLC7A11, or xCT, is to promote the body's synthesis of GSH, the main antioxidant. It achieves this by facilitating cystine absorption and glutamate release, thereby preserving the cellular redox balance, safeguarding cells against oxidative stress-induced harm, and preventing cell death triggered by lipid peroxidation [49-51].

Research has demonstrated that the SLC7A11 molecule is critical for the development, spread, metastasis, and resistance to several drugs for various types of cancers [10]. Sulfasalazine (SSZ), a potent and particular inhibitor of SLC7A11, can suppress tumor growth by inhibiting the Xc- system and reducing intracellular levels of GSH [52, 53]. SSZ can induce cystine/cysteine starvation, leading to glutathione depletion and significantly inhibiting the growth of prostate cancer DU145 and PC-3 cells [54], although the specific mechanisms involved remain unclear. Research on the involvement of SLC7A11 in the onset, progression, and therapeutic approaches for prostate cancer is still in its nascent stages. Earlier research has documented SLC7A11 to lead to the development of docetaxel resistance in PC. In this study, the c-Myc/miR-25-3p/SLC7A11 signaling axis has been found to be mediated by the Transcription Factor AP-2γ (TFAP2C), which can decrease ferroptosis in prostate cancer and promote chemoresistance [55]. Elevated levels of SLC7A11 are considered to be an unfavorable prognostic indicator for Overall Survival (OS) in 8 distinct forms of cancer, including adrenocortical carcinoma, PC, bladder cancer, and head and neck squamous cell carcinoma [56]. The results suggest a close correlation between SLC7A11 and the initiation, progression, and unfavorable prognosis of prostate cancer patients. SLC7A11 shows potential as a promising diagnostic and predictive marker for PC, as well as a viable candidate for therapy. However, the regulatory mechanisms upstream of SLC7A11 in the initiation and advancement of prostate cancer are still unclear and require further investigation.

The accumulation of lipid peroxides and oxidative stress characterizes ferroptosis. Alterations in Fe2+ and ROS levels can indirectly indicate the levels of oxidative stress and changes in ferroptosis. This study revealed an increase in Fe2+ and ROS levels in prostate cancer cells following circDUSP22 downregulation. This finding suggested that the downregulation of circDUSP22 enhances ferroptosis in prostate cancer cells, potentially serving as a significant factor in circDUSP22-mediated regulation of prostate cancer progression. The SLC7A11/GPX4 signaling axis is a pivotal mechanism that impedes ferroptosis. Our research has demonstrated that downregulating circDUSP22 can reduce the expression of SLC7A11 and GPX4 in PC cells, reduce the proliferative ability of cells, and increase the levels of Fe2+ and ROS in cells. These findings imply that circDUSP22 downregulation may cause ferroptosis in PC cells by blocking miR-18a-5p and altering the SLC7A11/GPX4 signaling pathway.

The therapeutic potential of ferroptosis has been recognized as a potential approach to cancer treatment [57-59]. Recent studies have identified sorafenib as an inducer of ferroptosis, with its cytotoxic effects on human cancer cells partially dependent on inducing ferroptosis in these cells [60]. By promoting sorafenib-induced ferroptosis, there is potential to enhance the efficacy of sorafenib treatment for hepatocellular carcinoma [61-65], gastric cancer [66], and clear cell renal cell carcinoma [67]. Currently, research has found circular RNA-mediated tumor ferroptosis to play a significant role in various cancers, such as lung cancer [47, 68-71], esophageal cancer [72], gastric cancer [73], colorectal cancer [74], pancreatic cancer [35], breast cancer [75, 76], cervical cancer [77, 78], renal cell carcinoma [79], bladder cancer [80], and thyroid cancer [21]. This study observed a significant increase in the expression of circDUSP22 in plasma samples from prostate cancer patients and cell lines, indicating its potential involvement in promoting ferroptosis in prostate cancer. The elevated expression of circDUSP22 in plasma may serve as a potential biomarker for diagnosing prostate cancer. However, this study lacks validation from animal models and large-scale multi-center clinical samples. The next steps for the research team will involve further animal model experiments and expanding sample sizes to validate the potential of circDUSP22 as both a therapeutic target and biomarker.

## CONCLUSION

**Fig. (11):**
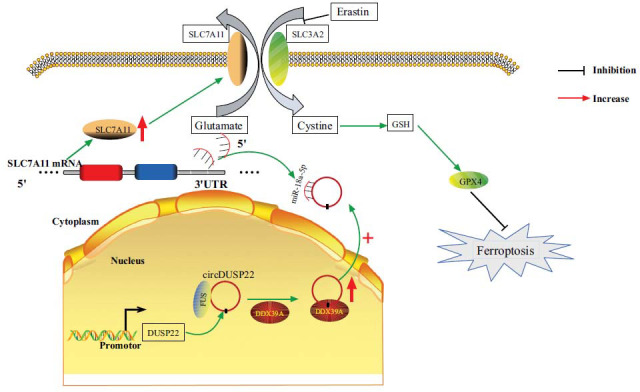
The molecular process *via* which circDUSP22 reduces the occurrence of ferroptosis in PC by modulating the miR-18a-5p/SLC7A11/GPX4 signaling pathway.

In summary, the elevated expression of circDUSP22 appears to promote cellular proliferation at the cellular level in prostate cancer. When circDUSP22 expression is suppressed, there is a significant decrease in the proliferative ability of prostate cancer cells. *In vitro* ferroptosis in prostate cancer cells has been demonstrated to be triggered by downregulation of circDUSP22; the mechanism of action is associated with the suppression of the SLC7A11/GPX4 signaling pathway (Fig. **11**). These findings serve as a foundation for investigating the process of circDUSP22 in PC and offer a pathway for molecular targeted therapy in PC.

## Data Availability

The data and supportive information are available within the article.

## STANDARDS OF REPORTING

STROBE guidelines were followed.

## References

[1] Bergengren O., Pekala K.R., Matsoukas K., Fainberg J., Mungovan S.F., Bratt O., Bray F., Brawley O., Luckenbaugh A.N., Mucci L., Morgan T.M., Carlsson S.V. (2023). 2022 Update on Prostate Cancer Epidemiology and Risk Factors—A Systematic Review.. Eur. Urol..

[2] Rosellini M., Santoni M., Mollica V., Rizzo A., Cimadamore A., Scarpelli M., Storti N., Battelli N., Montironi R., Massari F. (2021). Treating Prostate Cancer by Antibody–Drug Conjugates.. Int. J. Mol. Sci..

[3] Rizzo A., Mollica V., Tateo V., Tassinari E., Marchetti A., Rosellini M., De Luca R., Santoni M., Massari F. (2023). Hypertransaminasemia in cancer patients receiving immunotherapy and immune-based combinations: the MOUSEION-05 study.. Cancer Immunol. Immunother..

[4] Mollica V., Rizzo A., Rosellini M., Marchetti A., Ricci A.D., Cimadamore A., Scarpelli M., Bonucci C., Andrini E., Errani C., Santoni M., Montironi R., Massari F. (2021). Bone Targeting Agents in Patients with Metastatic Prostate Cancer: State of the Art.. Cancers (Basel).

[5] Rizzo A., Santoni M., Mollica V., Fiorentino M., Brandi G., Massari F. (2022). Microbiota and prostate cancer.. Semin. Cancer Biol..

[6] Wang Y., Ma Y., Jiang K. (2023). The role of ferroptosis in prostate cancer: a novel therapeutic strategy.. Prostate Cancer Prostatic Dis..

[7] Lee H., Zhuang L., Gan B. (2020). Energy stress inhibits ferroptosis *via* AMPK.. Mol. Cell. Oncol..

[8] Bertolio R., Napoletano F., Mano M., Maurer-Stroh S., Fantuz M., Zannini A., Bicciato S., Sorrentino G., Del Sal G. (2019). Sterol regulatory element binding protein 1 couples mechanical cues and lipid metabolism.. Nat. Commun..

[9] Wu J., Minikes A.M., Gao M., Bian H., Li Y., Stockwell B.R., Chen Z.N., Jiang X. (2019). Intercellular interaction dictates cancer cell ferroptosis *via* NF2–YAP signalling.. Nature.

[10] Koppula P., Zhang Y., Zhuang L., Gan B. (2018). Amino acid transporter SLC7A11/xCT at the crossroads of regulating redox homeostasis and nutrient dependency of cancer.. Cancer Commun..

[11] Koppula P., Zhuang L., Gan B. (2021). Cystine transporter SLC7A11/xCT in cancer: ferroptosis, nutrient dependency, and cancer therapy.. Protein Cell.

[12] Xia C., Xing X., Zhang W., Wang Y., Jin X., Wang Y., Tian M., Ba X., Hao F. (2024). Cysteine and homocysteine can be exploited by GPX4 in ferroptosis inhibition independent of GSH synthesis.. Redox Biol..

[13] Homma T., Kobayashi S., Fujii J. (2022). Methionine deprivation reveals the pivotal roles of cell cycle progression in ferroptosis that is induced by cysteine starvation.. Cells.

[14] Tang X., Chen W., Liu H., Liu N., Chen D., Tian D., Wang J. (2021). Research progress on SLC7A11 in the regulation of cystine/cysteine metabolism in tumors (Review).. Oncol. Lett..

[15] Yan Y., Teng H., Hang Q., Kondiparthi L., Lei G., Horbath A., Liu X., Mao C., Wu S., Zhuang L., James You M., Poyurovsky M.V., Ma L., Olszewski K., Gan B. (2023). SLC7A11 expression level dictates differential responses to oxidative stress in cancer cells.. Nat. Commun..

[16] Koppula P., Zhang Y., Shi J., Li W., Gan B. (2017). The glutamate/cystine antiporter SLC7A11/xCT enhances cancer cell dependency on glucose by exporting glutamate.. J. Biol. Chem..

[17] He F., Zhang P., Liu J., Wang R., Kaufman R.J., Yaden B.C., Karin M. (2023). ATF4 suppresses hepatocarcinogenesis by inducing SLC7A11 (xCT) to block stress-related ferroptosis.. J. Hepatol..

[18] Liu X., Chen C., Han D., Zhou W., Cui Y., Tang X., Xiao C., Wang Y., Gao Y. (2022). SLC7A11/GPX4 inactivation-mediated ferroptosis contributes to the pathogenesis of triptolide-induced cardiotoxicity.. Oxid. Med. Cell. Longev..

[19] Huang W., Chen K., Lu Y., Zhang D., Cheng Y., Li L., Huang W., He G., Liao H., Cai L., Tang Y., Zhao L., Pan M. (2021). ABCC5 facilitates the acquired resistance of sorafenib through the inhibition of SLC7A11-induced ferroptosis in hepatocellular carcinoma.. Neoplasia.

[20] Yadav P., Sharma P., Sundaram S., Venkatraman G., Bera A.K., Karunagaran D. (2021). SLC7A11/xCT is a target of miR-5096 and its restoration partially rescues miR-5096-mediated ferroptosis and anti-tumor effects in human breast cancer cells.. Cancer Lett..

[21] Wang H.H., Ma J.N., Zhan X.R. (2021). Circular RNA Circ_0067934 attenuates ferroptosis of thyroid cancer cells by miR-545-3p/SLC7A11 signaling.. Front. Endocrinol..

[22] Lang X., Green M.D., Wang W., Yu J., Choi J.E., Jiang L., Liao P., Zhou J., Zhang Q., Dow A., Saripalli A.L., Kryczek I., Wei S., Szeliga W., Vatan L., Stone E.M., Georgiou G., Cieslik M., Wahl D.R., Morgan M.A., Chinnaiyan A.M., Lawrence T.S., Zou W. (2019). Radiotherapy and immunotherapy promote tumoral lipid oxidation and ferroptosis *via* synergistic repression of SLC7A11.. Cancer Discov..

[23] Chen L., Wang C., Sun H., Wang J., Liang Y., Wang Y., Wong G. (2021). The bioinformatics toolbox for circRNA discovery and analysis.. Brief. Bioinform..

[24] Dong J., Zeng Z., Huang Y., Chen C., Cheng Z., Zhu Q. (2023). Challenges and opportunities for circRNA identification and delivery.. Crit. Rev. Biochem. Mol. Biol..

[25] Yu Y.Z., Lv D.J., Wang C., Song X.L., Xie T., Wang T., Li Z.M., Guo J.D., Fu D.J., Li K.J., Wu D.L., Chan F.L., Feng N.H., Chen Z.S., Zhao S.C. (2022). Hsa_circ_0003258 promotes prostate cancer metastasis by complexing with IGF2BP3 and sponging miR-653-5p.. Mol. Cancer.

[26] Pan J., Liu Z., Yang Z., Liang E., Fang C., Zhang D., Zhou X., Niu Y., Xin Z., Chen Y., Cai Q. (2022). Circ_0001686 Promotes Prostate Cancer Progression by Up-Regulating SMAD3/TGFBR2 *via* miR-411-5p.. World J. Mens Health.

[27] Zhang G., Liu Y., Yang J., Wang H., Xing Z. (2022). Inhibition of circ_0081234 reduces prostate cancer tumor growth and metastasis *via* the miR‐1/MAP 3 K1 axis.. J. Gene Med..

[28] Li W., Wu W. (2023). Circ_0005276 Promotes Prostate Cancer Progression Through the Crosstalk of miR-128-3p/DEPDC1B Axis.. Biochem. Genet..

[29] Ding X., Sun J., Zhang X. (2022). Circ_0076305 facilitates prostate cancer development *via* sponging miR‐411‐5p and regulating PGK1.. Andrologia.

[30] Lv D., Cen S., Yang S., Zou Z., Zhong J., Pan Z., Deng N., Li Y., Wu K., Wang J., Liu P. (2023). Hsa_circ_0063329 inhibits prostate cancer growth and metastasis by modulating the miR-605-5p/tgif2 axis.. Cell Cycle.

[31] Yu T., Du H., Sun C. (2023). Circ-ABCC4 contributes to prostate cancer progression and radioresistance by mediating miR-1253/SOX4 cascade.. Anticancer Drugs.

[32] Chen L., Song Y., Hou T., Li X., Cheng L., Li Y., Xing Y. (2022). Circ_0004087 interaction with SND1 promotes docetaxel resistance in prostate cancer by boosting the mitosis error correction mechanism.. J. Exp. Clin. Cancer Res..

[33] Zhao S., Li P., Wu W., Wang Q., Qian B., Li X., Shen M. (2021). Roles of ferroptosis in urologic malignancies.. Cancer Cell Int..

[34] Zhou X., Zou L., Liao H., Luo J., Yang T., Wu J., Chen W., Wu K., Cen S., Lv D., Shu F., Yang Y., Li C., Li B., Mao X. (2022). Abrogation of HnRNP L enhances anti-PD-1 therapy efficacy *via* diminishing PD-L1 and promoting CD8+ T cell-mediated ferroptosis in castration-resistant prostate cancer.. Acta Pharm. Sin. B.

[35] Liu T., Xie X., He Y., Zhang J., Mou J. (2024). circ_WASF2 regulates ferroptosis by miR-634/GPX4 signaling in pancreatic cancer.. Discov. Oncol..

[36] Huang X., Wu J., Wang Y., Xian Z., Li J., Qiu N., Li H. (2023). FOXQ1 inhibits breast cancer ferroptosis and progression *via* the circ_0000643/miR-153/SLC7A11 axis.. Exp. Cell Res..

[37] Huang M., Gao T., Chen X., Yi J., Zhou X. (2024). Circ_0087851 suppresses colorectal cancer malignant progression through triggering miR-593-3p/BAP1-mediated ferroptosis.. J. Cancer Res. Clin. Oncol..

[38] Wei L., He W., Zhao H., Zhao P. (2022). Circ_0026123 promotes cisplatin resistance and progression of ovarian cancer by upregulating RAB1A through sequestering miR-543.. Anticancer Drugs.

[39] Yamada M., Tanaka K., Yamamoto K., Matsumoto H., Yamasaki M., Yamashita K., Makino T., Saito T., Yamamoto K., Takahashi T., Kurokawa Y., Nakajima K., Okada Y., Eguchi H., Doki Y. (2023). Association between circ_0004365 and cisplatin resistance in esophageal squamous cell carcinoma.. Oncol. Lett..

[40] Shi W., Wang F. (2022). circ_AKT3 knockdown suppresses cisplatin resistance in gastric cancer.. Open Med..

[41] Ma T., Guo J., Han J., Li L., Ren Y., Huang J., Diao G., Zheng X., Zheng Y. (2023). Circ_0001589/miR‐1248/HMGB1 axis enhances EMT‐mediated metastasis and cisplatin resistance in cervical cancer.. Mol. Carcinog..

[42] Ma Y., Gao J., Guo H. (2023). Circ_0000140 Alters miR-527/SLC7A11-Mediated Ferroptosis to Influence Oral Squamous Cell Carcinoma Cell Resistance to DDP.. Pharm. Genomics Pers. Med..

[43] Wang Z., Ding Y., Wang X., Lu S., Wang C., He C., Wang L., Piao M., Chi G., Luo Y., Ge P. (2018). Pseudolaric acid B triggers ferroptosis in glioma cells *via* activation of Nox4 and inhibition of xCT.. Cancer Lett..

[44] Mishchenko T.A., Balalaeva I.V., Vedunova M.V., Krysko D.V. (2021). Ferroptosis and Photodynamic Therapy Synergism: Enhancing Anticancer Treatment.. Trends Cancer.

[45] Zhuo S., Yang L., Chen S., Tang C., Li W., Gao Z., Feng J., Yang K. (2022). Ferroptosis: A potential opportunity for intervention of pre-metastatic niche.. Front. Oncol..

[46] Lei G., Zhuang L., Gan B. (2024). The roles of ferroptosis in cancer: Tumor suppression, tumor microenvironment, and therapeutic interventions.. Cancer Cell.

[47] Li Z., Fan M., Zhou Z., Sang X. (2024). Circ_0082374 Promotes the Tumorigenesis and Suppresses Ferroptosis in Non-small Cell Lung Cancer by Up-Regulating GPX4 Through Sequestering miR-491-5p.. Mol. Biotechnol..

[48] Fantone S., Piani F., Olivieri F., Rippo M.R., Sirico A., Di Simone N., Marzioni D., Tossetta G. (2024). Role of SLC7A11/xCT in Ovarian Cancer.. Int. J. Mol. Sci..

[49] Chen R-S., Song Y-M., Zhou Z-Y., Tong T., Li Y., Fu M., Guo X-L., Dong L-J., He X., Qiao H-X., Zhan Q-M., Li W. (2009). Disruption of xCT inhibits cancer cell metastasis *via* the caveolin-1/β-catenin pathway.. Oncogene.

[50] Banjac A., Perisic T., Sato H., Seiler A., Bannai S., Weiss N., Kölle P., Tschoep K., Issels R.D., Daniel P.T., Conrad M., Bornkamm G.W. (2008). The cystine/cysteine cycle: A redox cycle regulating susceptibility versus resistance to cell death.. Oncogene.

[51] Huang Y., Dai Z., Barbacioru C., Sadée W. (2005). Cystine-glutamate transporter SLC7A11 in cancer chemosensitivity and chemoresistance.. Cancer Res..

[52] Guan J., Lo M., Dockery P., Mahon S., Karp C.M., Buckley A.R., Lam S., Gout P.W., Wang Y.Z. (2009). The x c − cystine/glutamate antiporter as a potential therapeutic target for small-cell lung cancer: Use of sulfasalazine.. Cancer Chemother. Pharmacol..

[53] Ma M., Chen G., Wang P., Lu W., Zhu C., Song M., Yang J., Wen S., Xu R., Hu Y., Huang P. (2015). Xc− inhibitor sulfasalazine sensitizes colorectal cancer to cisplatin by a GSH-dependent mechanism.. Cancer Lett..

[54] Doxsee D.W., Gout P.W., Kurita T., Lo M., Buckley A.R., Wang Y., Xue H., Karp C.M., Cutz J.C., Cunha G.R., Wang Y.Z. (2007). Sulfasalazine‐induced cystine starvation: Potential use for prostate cancer therapy.. Prostate.

[55] Jiang X., Guo S., Xu M., Ma B., Liu R., Xu Y., Zhang Y. (2022). TFAP2C-Mediated lncRNA PCAT1 Inhibits Ferroptosis in Docetaxel-Resistant Prostate Cancer Through c-Myc/miR-25-3p/SLC7A11 Signaling.. Front. Oncol..

[56] He J., Ding H., Li H., Pan Z., Chen Q. (2021). Intra-Tumoral Expression of SLC7A11 Is Associated with Immune Microenvironment, Drug Resistance, and Prognosis in Cancers: A Pan-Cancer Analysis.. Front. Genet..

[57] Bano I., Horky P., Abbas S.Q., Majid M., Bilal A.H.M., Ali F., Behl T., Hassan S.S., Bungau S. (2022). Ferroptosis: A New Road towards Cancer Management.. Molecules.

[58] Hassannia B., Vandenabeele P., Vanden Berghe T. (2019). Targeting Ferroptosis to Iron Out Cancer.. Cancer Cell.

[59] Chen X., Kang R., Kroemer G., Tang D. (2021). Broadening horizons: the role of ferroptosis in cancer.. Nat. Rev. Clin. Oncol..

[60] Li Q., Chen K., Zhang T., Jiang D., Chen L., Jiang J., Zhang C., Li S. (2023). Understanding sorafenib-induced ferroptosis and resistance mechanisms: Implications for cancer therapy.. Eur. J. Pharmacol..

[61] Yang C., Lu T., Liu M., Yuan X., Li D., Zhang J., Zhou L., Xu M. (2023). Tiliroside targets TBK1 to induce ferroptosis and sensitize hepatocellular carcinoma to sorafenib.. Phytomedicine.

[62] Byun J.K., Lee S., Kang G.W., Lee Y.R., Park S.Y., Song I.S., Yun J.W., Lee J., Choi Y.K., Park K.G. (2022). Macropinocytosis is an alternative pathway of cysteine acquisition and mitigates sorafenib-induced ferroptosis in hepatocellular carcinoma.. J. Exp. Clin. Cancer Res..

[63] Gao R., Kalathur R.K.R., Coto-Llerena M., Ercan C., Buechel D., Shuang S., Piscuoglio S., Dill M.T., Camargo F.D., Christofori G., Tang F. (2021). YAP/TAZ and ATF4 drive resistance to Sorafenib in hepatocellular carcinoma by preventing ferroptosis.. EMBO Mol. Med..

[64] Liu M., Shi C., Song Q., Kang M., Jiang X., Liu H., Pei D. (2023). Sorafenib induces ferroptosis by promoting TRIM54-mediated FSP1 ubiquitination and degradation in hepatocellular carcinoma.. Hepatol. Commun..

[65] Xu F., Wu X., Chen C., Wang K., Huang L., Xia J., Liu Y., Shan X., Tang N. (2023). SLC27A5 promotes sorafenib-induced ferroptosis in hepatocellular carcinoma by downregulating glutathione reductase.. Cell Death Dis..

[66] Xu X., Li Y., Wu Y., Wang M., Lu Y., Fang Z., Wang H., Li Y. (2023). Increased ATF2 expression predicts poor prognosis and inhibits sorafenib-induced ferroptosis in gastric cancer.. Redox Biol..

[67] Chang K., Chen Y., Zhang X., Zhang W., Xu N., Zeng B., Wang Y., Feng T., Dai B., Xu F., Ye D., Wang C. (2023). DPP9 Stabilizes NRF2 to Suppress Ferroptosis and Induce Sorafenib Resistance in Clear Cell Renal Cell Carcinoma.. Cancer Res..

[68] Zhao Y., Cui Q., Shen J., Shen W., Weng Y. (2023). Hsa_circ_0070440 promotes lung adenocarcinoma progression by SLC7A11-mediated-ferroptosis.. Histol. Histopathol..

[69] Pan C.F., Wei K., Ma Z.J., He Y.Z., Huang J.J., Guo Z.Z., Chen Z.P., Barr M.P., Shackelford R.E., Xia Y., Wang J. (2022). CircP4HB regulates ferroptosis *via* SLC7A11-mediated glutathione synthesis in lung adenocarcinoma.. Transl. Lung Cancer Res..

[70] Shanshan W., Hongying M., Jingjing F., Yiming Y., Yu R., Rui Y. (2021). CircDTL Functions as an Oncogene and Regulates Both Apoptosis and Ferroptosis in Non-small Cell Lung Cancer Cells.. Front. Genet..

[71] Liu B., Ma H., Liu X., Xing W. (2023). CircSCN8A suppresses malignant progression and induces ferroptosis in non-small cell lung cancer by regulating miR-1290/ACSL4 axis.. Cell Cycle.

[72] Xi Y., Shen Y., Wu D., Zhang J., Lin C., Wang L., Yu C., Yu B., Shen W. (2022). CircBCAR3 accelerates esophageal cancer tumorigenesis and metastasis *via* sponging miR-27a-3p.. Mol. Cancer.

[73] Li C., Tian Y., Liang Y., Li Q. (2020). RETRACTED ARTICLE: Circ_0008035 contributes to cell proliferation and inhibits apoptosis and ferroptosis in gastric cancer *via* miR-599/EIF4A1 axis.. Cancer Cell Int..

[74] Wang Y., Chen H., Wei X. (2021). Circ_0007142 downregulates miR‐874‐3p‐mediated GDPD5 on colorectal cancer cells.. Eur. J. Clin. Invest..

[75] Zhang H., Ge Z., Wang Z., Gao Y., Wang Y., Qu X. (2021). Circular RNA RHOT1 promotes progression and inhibits ferroptosis *via* mir-106a-5p/STAT3 axis in breast cancer.. Aging (Albany NY).

[76] Wang S., Wang Y., Li Q., Li X., Feng X. (2022). A novel circular RNA confers trastuzumab resistance in human epidermal growth factor receptor 2‐positive breast cancer through regulating ferroptosis.. Environ. Toxicol..

[77] Wu P., Li C., Ye D., Yu K., Li Y., Tang H., Xu G., Yi S., Zhang Z. (2021). Circular RNA circEPSTI1 accelerates cervical cancer progression *via* miR-375/409-3P/515-5p-SLC7A11 axis.. Aging (Albany NY).

[78] Ou R., Lu S., Wang L., Wang Y., Lv M., Li T., Xu Y., Lu J., Ge R. (2022). Circular RNA circLMO1 Suppresses Cervical Cancer Growth and Metastasis by Triggering miR-4291/ACSL4-Mediated Ferroptosis.. Front. Oncol..

[79] Cen J., Liang Y., Feng Z., Chen X., Chen J., Wang Y., Zhu J., Xu Q., Shu G., Zheng W., Liang H., Wang Z., Deng Q., Cao J., Luo J., Jin X., Huang Y. (2023). Hsa_circ_0057105 modulates a balance of epithelial‐mesenchymal transition and ferroptosis vulnerability in renal cell carcinoma.. Clin. Transl. Med..

[80] Wang L., Wu S., He H., Ai K., Xu R., Zhang L., Zhu X. (2022). CircRNA-ST6GALNAC6 increases the sensitivity of bladder cancer cells to erastin-induced ferroptosis by regulating the HSPB1/P38 axis.. Lab. Invest..

